# The Sequential Aerosol Technique: A Major Component in an Integrated Strategy of Intervention against Riverine Tsetse in Ghana

**DOI:** 10.1371/journal.pntd.0002135

**Published:** 2013-03-14

**Authors:** Yahaya Adam, Giuliano Cecchi, Patrick M. Kgori, Tanguy Marcotty, Charles I. Mahama, Martin Abavana, Benita Anderson, Massimo Paone, Raffaele Mattioli, Jérémy Bouyer

**Affiliations:** 1 Veterinary Services Department of the Ministry of Food and Agriculture, Pong-Tamale, Ghana; 2 Food and Agriculture Organization of the United Nations, Animal Production and Health Division, Rome, Italy; 3 Ministry of Agriculture, Department of Veterinary Services, Maun, Botswana; 4 Department of Biomedical Sciences, Institute of Tropical Medicine, Antwerpen, Belgium; 5 Department of Veterinary Tropical Diseases, Faculty of Veterinary Science, University of Pretoria, Pretoria, South Africa; 6 Veterinary Services Department of the Ministry of Food and Agriculture, Accra, Ghana; 7 Unité Mixte de Recherche Contrôles des Maladies Animales et Emergentes, Centre de Coopération Internationale en Recherche Agronomique pour le Développement, Montpellier, France; 8 Institut Sénégalais de Recherches Agricoles, Laboratoire National d'Elevage et de Recherches Vétérinaires, Dakar, Sénégal; National Institute of Allergy and Infectious Diseases, United States of America

## Abstract

**Background:**

An integrated strategy of intervention against tsetse flies was implemented in the Upper West Region of Ghana (9.62°–11.00° N, 1.40°–2.76° W), covering an area of ≈18,000 km^2^ within the framework of the Pan-African Tsetse and Trypanosomosis Eradication Campaign. Two species were targeted: *Glossina tachinoides* and *Glossina palpalis gambiensis*.

**Methodology/Principal Findings:**

The objectives were to test the potentiality of the sequential aerosol technique (SAT) to eliminate riverine tsetse species in a challenging subsection (dense tree canopy and high tsetse densities) of the total sprayed area (6,745 km^2^) and the subsequent efficacy of an integrated strategy including ground spraying (≈100 km^2^), insecticide treated targets (20,000) and insecticide treated cattle (45,000) in sustaining the results of tsetse suppression in the whole intervention area. The aerial application of low-dosage deltamethrin aerosols (0.33–0.35 g a.i/ha) was conducted along the three main rivers using five custom designed fixed-wings Turbo thrush aircraft. The impact of SAT on tsetse densities was monitored using 30 biconical traps deployed from two weeks before until two weeks after the operations. Results of the SAT monitoring indicated an overall reduction rate of 98% (from a pre-intervention mean apparent density per trap per day (ADT) of 16.7 to 0.3 at the end of the fourth and last cycle). One year after the SAT operations, a second survey using 200 biconical traps set in 20 sites during 3 weeks was conducted throughout the intervention area to measure the impact of the integrated control strategy. Both target species were still detected, albeit at very low densities (ADT of 0.27 inside sprayed blocks and 0.10 outside sprayed blocks).

**Conclusions/Significance:**

The SAT operations failed to achieve elimination in the monitored section, but the subsequent integrated strategy maintained high levels of suppression throughout the intervention area, which will contribute to improving animal health, increasing animal production and fostering food security.

## Author Summary

We document the impact of an integrated strategy of intervention against riverine tsetse flies in the Upper West Region of Ghana within the framework of the Pan-African Tsetse and Trypanosomosis Eradication Campaign, in an area of ≈18,000 km^2^. The strategy included a sequential aerosol technique (SAT) component, i.e. four applications of low-dosage deltamethrin aerosols, conducted along the three main rivers. The impact of SAT on tsetse densities was monitored in a challenging subsection (dense tree canopy and high tsetse densities) from two weeks before until two weeks after the operations. The SAT operations succeeded in reducing tsetse populations by 98% within one month but fell short of achieving elimination. Insecticide ground spraying, deltamethrin-treated targets and cattle were used as complementary tools to maintain tsetse suppression in the intervention area. An entomological survey conducted one year after SAT operations showed that both target species were still present, albeit at drastically reduced densities as compared to the baseline levels. This integrated strategy of intervention will contribute to improving animal health, increasing animal production and fostering food security in the target area.

## Introduction

In sub-Saharan Africa the tsetse fly (Genus: *Glossina*) is the cyclical vector of trypanosomosis, a disease of livestock and humans, caused by unicellular parasites of the genus *Trypanosoma*. Whilst the human form of the disease no longer appears to be a major public health issue in Ghana [1]–[3], animal trypanosomosis is still widely reported and causes considerable losses in the livestock sector resulting in major impacts on agricultural production, livelihoods and food security [4], [5].

In Ghana, *Glossina* species have raised concern from the beginning of the colonial period [6]. Since then, different techniques have been used in a number of control efforts [7]–[9]. In Ghana, these techniques included, *inter alia*, the removal of tsetse habitat by bush clearing, the destruction of wildlife, as well as the use of insecticide treated targets (ITT) and insecticide treated cattle (ITC) [5], [10]–[12]. These techniques, albeit successful in suppressing tsetse populations, failed to eliminate the tsetse fly from Ghana [5]. In this paper, the term ‘elimination’ is used to designate a local eradication.

In the control area, located in the Upper West Region, *G. tachinoides* and *G. palpalis gambiensis* are the sole species present [5], [13]. *G. morsitans submorsitans* is absent, but found at 30 km only north-east to the target area, in the Sissili protected forest. Both *G. tachinoides* and *G. palpalis gambiensis* are riverine species, for which riparian vegetation represents the typical habitat [14]–[16]. In the study area, this type of habitat is mainly found along the rivers Black Volta, Kulpawn and Sissili. All of these rivers flow southwards into Lake Volta, but parts of their catchment basins are located to the North, in neighbouring Burkina Faso. Along the tributaries of the three main rivers, the habitat appears unsuitable for tsetse flies in the dry season (December–May), during which bush fires and the absence of rain seriously limit the availability of appropriate vegetation [13]. At this time of year, tsetse flies retreat from tributaries to the main rivers, where permanent water and dense vegetation mitigate the harsh ambient macroclimatic conditions (e.g. temperatures often rising above 40°C).

Large-scale aerial spraying of insecticide was adopted as the main intervention tool against tsetse flies in the Upper West Region of Ghana within the frame work of the African Union's Pan-African Tsetse and Trypanosomosis Eradication Campaign (PATTEC), whose ultimate goal is to eradicate African trypanosomosis through the progressive and sustainable creation of tsetse-and trypanosomosis-free areas [17]. In Ghana, an integrated strategy of intervention was adopted, whereby the sequential aerosol technique (SAT) was complemented by ground spraying, insecticide treated targets (ITT) and insecticide treated cattle (ITC). To promote sustainability and limit the risk of post-operation reinvasion, the integrated intervention strategy was based on regional collaboration. Contrary to previous control campaigns conducted at a local scale, SAT operations were conducted jointly in Ghana and Burkina Faso. The impact of the integrated interventions on non-target aquatic, terrestrial and insectivorous fauna was monitored and will be presented elsewhere.

The present study aimed at evaluating the potentiality of SAT to achieve elimination of the riverine tsetse species in a particularly challenging subsection (dense tree canopy and high tsetse densities as revealed by the baseline survey) and the subsequent efficacy of the integrated strategy including ground spraying, ITT and ITC to sustain the results of tsetse suppression in the Upper West Region of Ghana.

## Materials and Methods

### Study area and sequential aerosol technique

**Figure 1:**
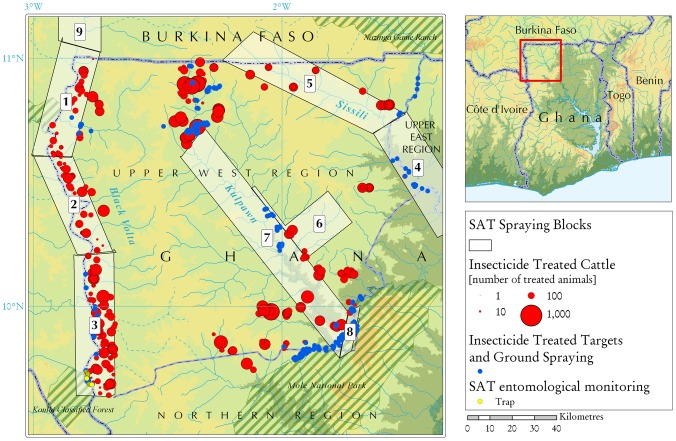
Tsetse control operations conducted in the Upper West Region of Ghana. The map represents the situation from March 2010 to December 2011.

The study area is located in north-western Ghana (from lat. 9.62 to 11.00 N and from long. 1.40 to 2.76 W), and it covers a surface of ≈18,000 km^2^ (Figure 1).

SAT, consisting of repeated spraying of non-residual insecticide aerosols from the air [18],was applied to the three main rivers (Black Volta, Sissili and Kulpawn). Eight spraying blocks of different size were defined (Figure 1), covering a total area of 6,745 km^2^. A ninth block of 1,940 km^2^ was located across the border in Burkina Faso. A 15 km blocks' width assured the coverage of the tsetse-infested riparian vegetation, whilst the block lengths were selected to allow parallel flying lines.

The aerial application of low-dosage insecticide aerosols was conducted following the principles described by Kgori *et al.* (2006). The insecticide application rates were adjusted to suit local climatic conditions, and 4 cycles were used (as opposed to the 5 conducted in Botswana in the operations described by Kgori *et al.* (2006)). Air-spraying was conducted at night, along swaths of 275 m width. Occasionally, daytime spraying was carried out under stable and cool overcast weather conditions when suitable temperature inversion was recorded. Such modification of the standard SAT procedure was necessary to maintain continuity of the spraying programme following, for instance, unavoidable interruptions due to inappropriate weather conditions. The operation was carried out by Orsmond Aviation (Bethlehem, South Africa), using five custom designed fixed-wing Turbo thrush aircraft – fitted with navigation and spray management equipment.

The applied insecticide was Deltamethrin (0.35% (w/v), ultra-low volume (ULV) (Deltanex formulation, Avima, Johannesburg, South Africa).The insecticide dispersal units, fitted to each aircraft were made of two boom-mounted, wind-driven Micron-air AU 4000 rotary atomisers (Micron Sprayers Ltd., Bromyard. UK) operated with cage speed of 11,000rpm and average flow rate of 9.7 l/km^2^. The micron-airs were fitted with shut-off valves to prevent spillage in the event of damage to the atomizers. Each aircraft was also fitted with the upgraded GPS-based guidance system - SATLOC M3 (CSI Wireless, Calgary, Canada), to provide precision controlled spray application aided by a moving map display that guided the pilot using an external light-bar. Flight and spraying statistics were captured automatically using the system's integrated data logger [19].

The spectrum of aerosol droplets was assessed using magnesium oxide coated glass slides. While rotating at 330 rpm, the Numerical and Volume Median Diameter values were 17.70 and 35.50 µm respectively. As the insecticide had been stored locally for about one year before usage, samples of the formulation were sent to a laboratory (Pesticide Analytical Technology, Pretoria, South Africa) for potency testing. Despite storage at high temperatures, both the physical and chemical properties of the product were confirmed to be within the expected ranges.

**Figure 2:**
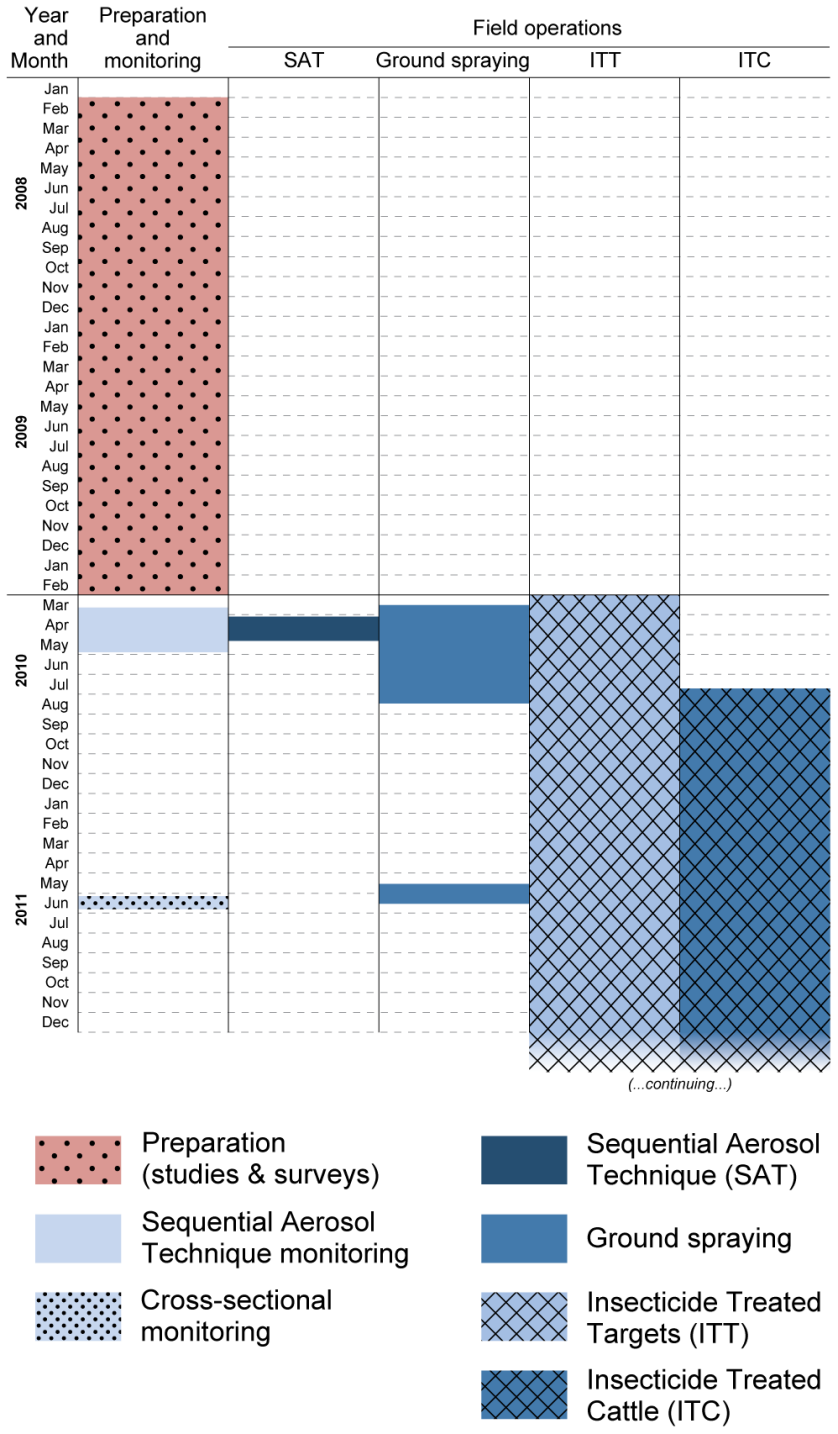
Timeline of the preparation and implementation of the integrated intervention strategy against riverine tsetse. For field operations the bars indicate the start and end date of deployment. More details on the spatial configuration and the frequency of deployment are provided in the text.

SAT operations were conducted between 3 April and 5 May 2010 (Figure 2). For the first spraying cycle, the application rate was at 0.33 g active ingredient (a.i)/ha in all the sprayed blocks. Subsequent treatments of blocks 3 and 7 used 0.35 g a.i/ha.

**Figure 3:**
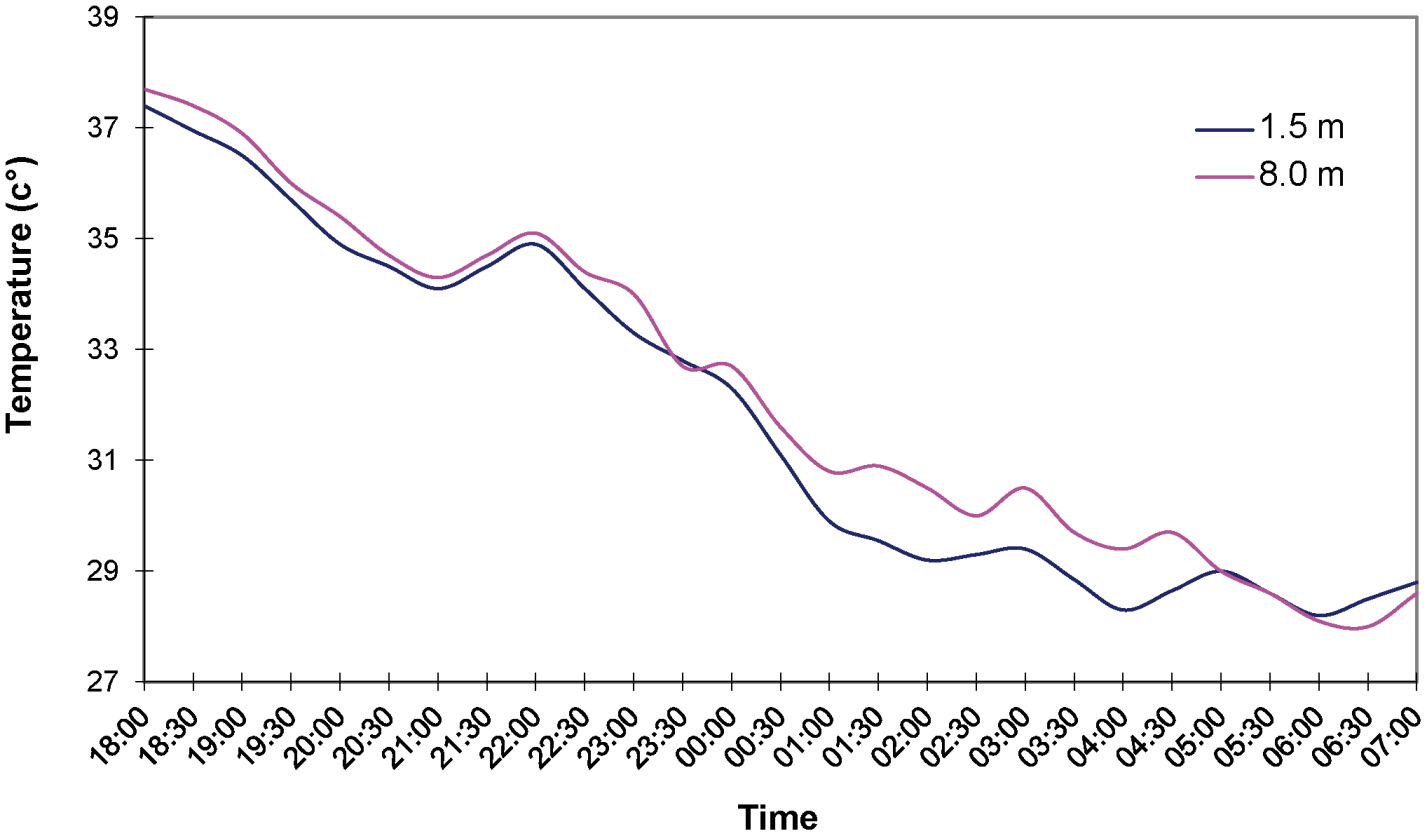
Temperatures inversion layer measured from Wa airport on 2 April 2010.

The choice of the spraying period, i.e. late dry season, as well as the timing, i.e. from dusk to dawn, aimed at optimising the advantage of temperature inversion conditions with gentle winds that allowed the smaller aerosol droplets to descend into tsetse habitat. Temperature inversion was investigated using probes located at a height of approximately 1.5 and 8 m. The inversion layer was shallow in the early afternoon, improving through the night and breaking down around 0700h in the morning (Figure 3). Flying height was about 10 m above tree canopy.

Localised areas of thick vegetation in blocks 3 and 7 presented serious challenges to insecticide penetration. Therefore, in those areas three supplementary swaths were added along the edges of the river to increase the application rate during the three last spraying cycles.

In SAT, the sequence of insecticide application must be timed so that female tsetse flies emerging after one treatment have no sufficient time to deposit larvae before the next treatment. This period is called the first larval period (FLP). In planning the inter-cycle period, estimates of FLP are to be taken into account. Moreover, the sequence of application has to continue until new adults cease appearing from underground, i.e. until the pupal period (PP) is completed. These two parameters are temperature dependent and can be calculated from the Jackson's formulae [20], where t is the temperature in degrees Celsius (°C):
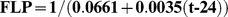


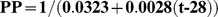



In the Upper West Region, the initial spraying schedule was based on a six-year average of daily temperatures at Wa, the regional capital. The estimated FLP and PP were 11 and 20 days respectively. Spraying began on 3 April 2010. Subsequent cycles started on 12April, 22April and 3 May. During operations, meteorological data were collected to control that planning was appropriate.

### Other control techniques

Figure 2 provides a timeline of the preparation and implementation of the integrated intervention strategy. Ground spraying of insecticide was one of the complementary control measures deployed after SAT operations in order to sustain the results of tsetse suppression in the intervention area. Ground spraying was conducted in the sites where the tree canopy was too dense to allow a sufficient penetration of the insecticide sprayed by air and where targets were placed to act as barriers against reinvasion from non-treated tsetse infested areas (Figure 1). Motorised hand sprayers were employed using Deltamethrin sc 20% (200 g/litre of water). A dose of 100 ml insecticidal concentrate per 18 litres of water (one knapsack sprayer) was used to obtain a concentrated spray liquid (0.1%), allowing chemical persistence at active levels for 7 months (FAO 1992). Apart from a core staff of 12 organized in 3 teams of 4 persons working simultaneously in different locations, each team recruited and trained ≈10 staff from the communities as casual labour for the ground spraying operations. Ground spraying was applied to tree trunks between and around targets, under overhanging rocks, branches and twigs that provide resting and breeding sites for tsetse. Ground spraying operations concerned ≈100 km^2^ (i.e. ≈5 km^2^ in the Black Volta river basin, ≈54 km^2^ in the Kulpawn River basin and ≈40 km^2^ in the Sissili River basin) and took place from March to August 2010 and from May to June 2011. To compensate for the inability to repeat the spraying more frequently, the Deltamethrin formulation used had an added UV inhibitor to extend the effective life of the insecticide.

Deltamethrin (1% w/v) pour-on formulation (Shandong Luxi Animal Medicine Company-P.R. China) was applied along the backline of cattle at a dose of 1 ml/10 kg of body weight. In selecting animals for pour-on treatment, priority was given to those located in the vicinity of rivers (Figure 1). All the animals of at least 50% and in most cases 100% of all kraals located within 10 km from one of the three main rivers were treated. Over 45,000 cattle were treated using approximately 454 litres of deltamethrin (1%) pour-on formulation. The project conducted the first pour on treatment (start date 22 July 2010) and distributed additional product to the farmers thanks to locally trained staff, so that they could conduct monthly treatments by themselves.

ITT were deployed from March 2010 in the intervention area at locations strategically selected to serve a dual purpose (Figure 1). First, targets were placed along sections of the perimeter of the intervention area, especially at river crossings and at the border with game reserves, in an effort to stem reinvasion from non-treated areas. Second, ITT were deployed both within and outside SAT-treated blocks to act as a complementary suppression tool. The density of ITT was greater at the limit of the Mole National Park, where tsetse densities and reinvasion pressure are at their highest. The targets corresponded to PermaNet 2.0^ND^, comprising 100% polyester panels, sized W150×H100 cm (Vestergard Frandsen Group) and with a central blue panel flanked by black side panels of 50 cm wide each.

Targets were placed in four rows spaced by 50 m, in order to form 150 m-deep barriers along protected areas, main rivers and their tributaries. Horizontal target-spacing was 25 m for the first row, closest to the potential source of reinvasion, and 50 m for the other rows. Overall, about 20,000 deltamethrin treated targets were deployed on a total linear distance of about 89 km.

### Monitoring the impact of the control campaign

The impact of the different SAT cycles on tsetse population density was monitored in one site only, at the southernmost part of the study area in Block3. The site was considered to be particularly challenging for SAT because of its dense vegetation and the high density of tsetse [13]. Monitoring in the other spraying blocks could not be conducted because of budgetary and logistical constraints. Thirty biconical traps [21] were deployed with an average inter-trap distance of 200 meters. Traps were deployed from two weeks before and until two weeks after the four spraying cycles. Fly catches were collected and sorted on a daily basis. Fly species, sex and age were recorded. Age was only estimated in females through ovarian dissection and observation [22]. Catches were analysed using a robust negative binomial regression in Stata 11. Individual traps were considered as primary sampling units whereas post SAT periods were used as categorical explanatory variables. Suppression rates were estimated for each SAT spraying cycle and for the four cycles as a whole.

From 6 to 24 June 2011, approximately one year after SAT operations, a cross-sectional entomological survey was conducted to determine the cumulative impact of the integrated control measures across the project area. Monitoring sites were selected taking into account (i) the spatial configuration of the integrated tsetse suppression operations (Figure 1), with particular attention to the SAT sprayed blocks, (ii) pre-operation apparent densities of tsetse flies and prevalence of bovine trypanosomosis [13], and (iii) the risk of fly reinvasion.

Guided by the above principles, and also using Landsat 7 Satellite images to identify potential tsetse habitat, 20 monitoring sites were selected. At each site, 10 biconical traps spaced 100 m were deployed. Traps were inspected twice a day for fly catches and removed after 48 hours of trapping. Flies caught were sorted by species, physiological status and sex. Data were analysed in a robust negative binomial regressions using the sites as primary sampling units. The total abundance was first estimated as a function of the location (within or outside the SAT areas). Species abundance was estimated in a second model using species as categorical explanatory variable.

### Ethical statement

All necessary permits were obtained by the PATTEC/Ghana project, which is a national project of the Ministry of Food & Agriculture. For the observance of good ethics, the project collaborated with the Ghana Environmental Protection Agency, Wild Life Division and the Ghana Wild Life Society who monitored the exercise throughout the operational period. All individuals, communities and local authorities within the project area were sensitised and permission granted for the execution of the project.

## Results

### SAT schedule

**Figure 4:**
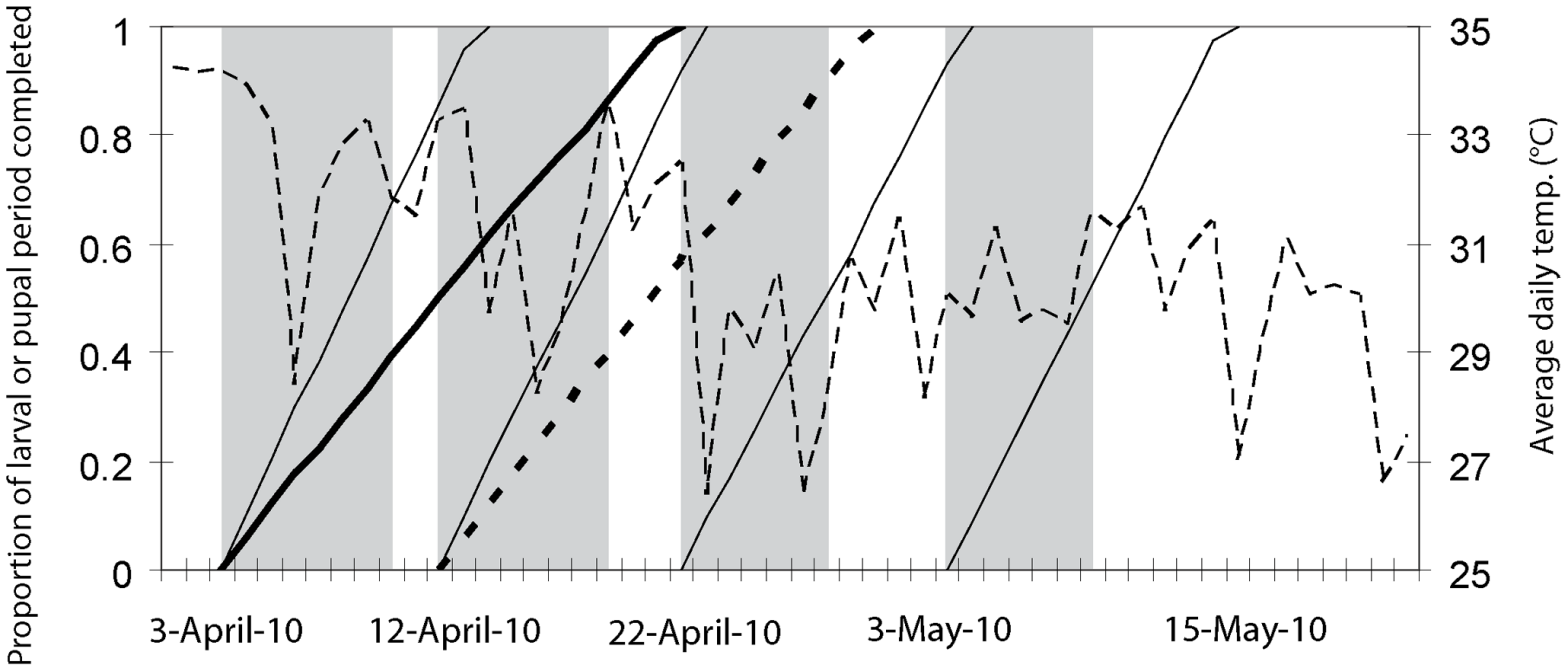
Course of larval and pupal developments durations during the SAT operations in Ghana. The thin solid lines present the larval development and the thick solid line the pupal development, estimated using Jackson's formulae (see (Hargrove, 2003)). Mean daily temperature is presented as a dashed line. A second pupal period (thick dashed line), starting at the beginning of the second cycle, shows the potential development of pupae produced by females surviving to the first treatment. Vertical bars indicate the periods of the four SAT applications.

According to local meteorological data measured during field operations, the SAT schedule fitted exactly the predicted FLP (Figure 4). More precisely, each cycle was conducted one to two days before the predicted days of larviposition of primipares. Moreover, the whole treatment period lasted much more than the maximum PP.

### Efficacy of the SAT cycles

**Figure 5:**
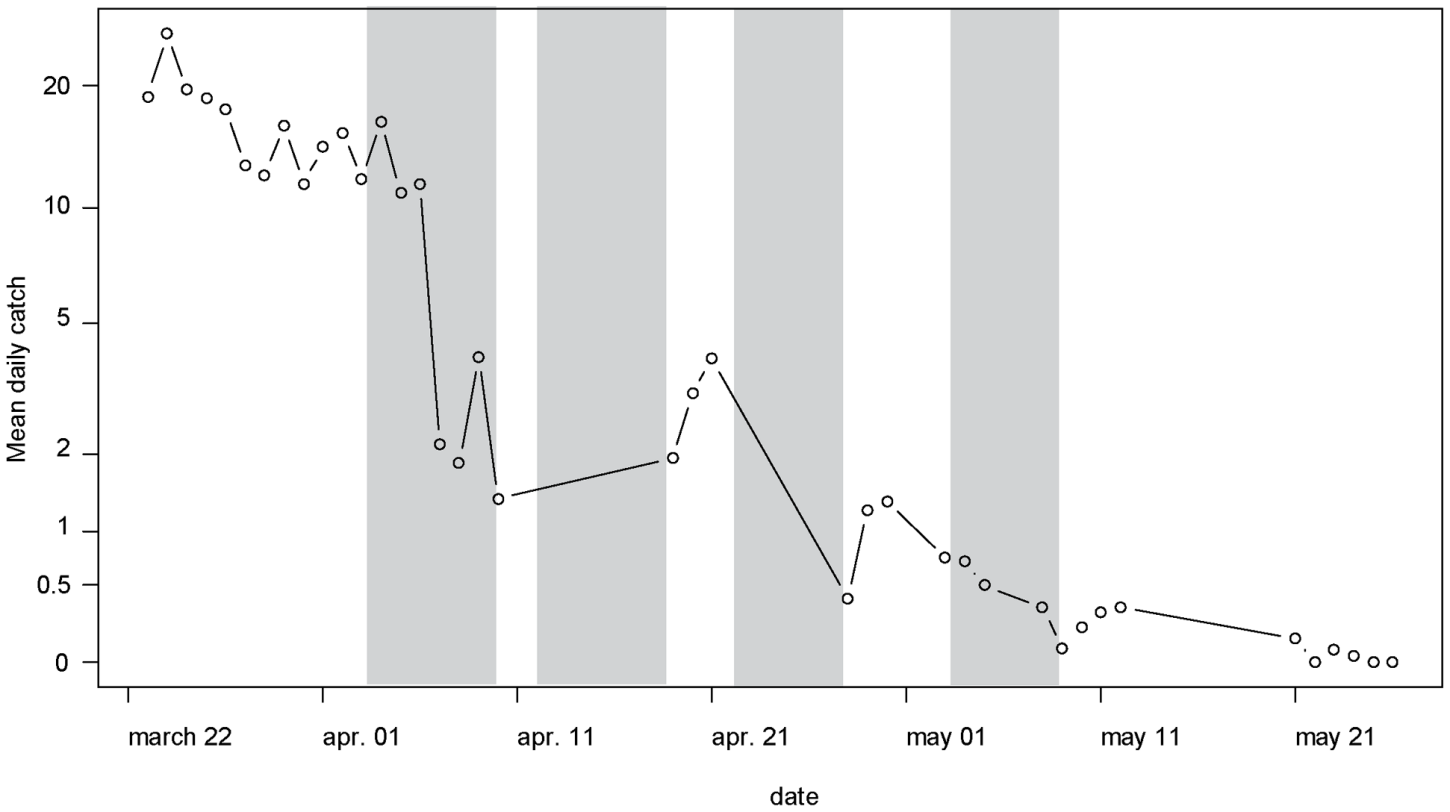
Mean daily catch of tsetse before and during the SAT operation in Ghana. Vertical bars indicate the periods of SAT applications.

In the densely vegetated site chosen for SAT monitoring, the mean apparent density per trap per day (ADT) dropped from 16.7 (95%CI10.4–26.8) before spraying to 1.4 (0.7–1.7), 3.6 (1.8–6.8), 1.3 (0.6–3.0) and 0.3 (0.1–0.5) during the two days immediately after spraying cycles 1 to 4 respectively. This corresponded to reduction rates of 92% (85–95%, p<0.001), 0% (a significant increase was even observed, p = 0.001), 64% (46–76%, p<0.001) and 78% (76–86%, p<0.001) respectively (Figure 5). The resulting overall reduction rate was 98% (97–99%, p<0.001). ADT further dropped to 0.04 (0.01–0.11) on the last observation week (12 to 19 days after the last spraying).

Tsetse dissection indicated a continuous reduction of non-teneral female flies from 1,223 flies before spraying to 8 flies after cycle 4 (Table 1). The percentage of non teneral flies declined from 63%(s.d5%) before SAT operations to 61%(s.d.6%), 41% (s.d.5%), 14%(s.d.6%) and 33%(s.d.14%) after cycles 1 to 4 respectively (X-squared = 34, df = 4, p<10^−3^). However, the amounts of non teneral flies proved that at least 22% of the adult females dissected after each cycle were survivors or immigrants. The emerging juveniles also declined from 726 to 17 after the last cycle.

**Table 1:** Tsetse catches during SAT operations.

		Dissected females		
Period	Total catch	Teneral	Non-teneral	Total
Before spraying	1949	35	59	94
Cycle 1	219	30	47	77
Cycle 2	271	57	39	96
Cycle 3	90	32	5	37
Cycle 4	25	8	4	12

The data correspond to the total tsetse catches, teneral, non-teneral and total dissected female flies before (2 weeks) and after each SAT cycle (2–3 days for cycles 1 to 3 and 2 weeks after cycle 4).

### Efficacy of the integrated tsetse control campaign

**Figure 6:**
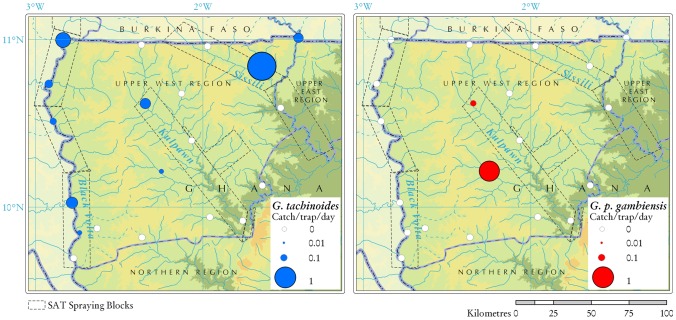
Results of the entomological survey conducted in Ghana one year after SAT operations. The survey was conducted from 6^th^ to 24th June 2011to monitor the impact of the integrated tsetse control campaign. Tsetse apparent density is expressed as the number of catches per trap per day.

One year after SAT operations, the apparent density of tsetse flies was still very low throughout the project area (Figure 6), with average ADT of 0.27 (95% CI: 0.1–0.7) inside the sprayed blocks, and 0.10 (95% CI: 0.02–0.5) outside the sprayed blocks. The difference was not significant (p = 0.35).

Rigorous comparisons with pre-intervention densities are difficult to make because of differences in the design of the baseline and post-intervention cross-sectional surveys. In terms of species relative abundance, post-intervention entomological data were in line with pre-operation surveys, in that *G. tachinoides* was more abundant and much more widespread than *G. palpalis gambiensis* (p = 0.18), with ADT of 0.17 (95% CI: 0.06–0.47) and 0.03 (95% CI: 0.005–0.2) respectively. Even after a year of the beginning of the integrated tsetse control, *G. tachinoides* was captured in all three river basins in the project area, and all along the Black Volta. The highest density was detected in one site along the river Sissili, where the complementary pressure from ITC, ITC and ground spraying was comparatively lower. *G. palpalis gambiensis* was captured in two sites within the Kulpawn river basin (including one site located along a right-hand tributary that lies outside the SAT sprayed blocks).

## Discussion

In southern Africa, SAT was successfully used to eliminate *G. morsitans centralis* from the Okavango Delta of Botswana, as confirmed by sustained post-intervention monitoring [18], [23]. In the Okavango Delta, climatic and biophysical conditions such as the presence of open savannah, together with the ecology of the target tsetse species, enhanced the impact of SAT. Two main lessons can be drawn from this study: first, in the Upper West Region of Ghana, SAT achieved very high levels of suppression in one of the most challenging sites, but failed to achieve elimination of the tsetse population. It must be acknowledged that the density of the vegetation was highest in this section, and that elimination might have been achieved in other sections of the target area, where the gallery is more open. The possibility of extrapolating the present results is therefore limited. However, in an elimination perspective, a partial failure hampers the results in the whole area, since tsetse can then re-invade the cleared areas. Second, the integrated strategy of intervention succeeded in sustaining the results of suppression one year after the SAT operations.

As the predicted FLP and PP used to schedule SAT matched the FLP and PP derived from the real temperature data measured during operations, it is unlikely that inability to eliminate tsetse be ascribable to inappropriate synchronisation of pupae sequestration in the soil and SAT application. Yet, FLP and PP were estimated using average climatic data and, as such, may not account for substantial micro-climatic heterogeneity found in riparian habitats [24], which are often characterized by various levels of disturbance and fragmentation of the natural vegetation [14], [25]. Moreover, we have no field data for the target species and Hargrove has pointed out a strong variability of FLP (up to 3 days) between individuals [20].

Arguably, the single most important factor affecting the efficacy of SAT operations in Ghana was the presence of patches of thick vegetation, which pose serious challenges to insecticide penetration into tsetse habitat. Attempts were made to measure droplet penetration both under the canopy and in the open during last cycle, but unfortunately the samples were damaged by excessive overnight dew and no detectable chemical was apparent in the results. Earlier experiences of aerial spraying against riverine tsetse in West Africa successfully addressed the problem of penetrating riverine forest by using helicopters, based on the assumption that the downdraft from the helicopters would help the insecticide penetrate beneath the canopy [26]–[28]. However, residual insecticides were used, with significant impacts on non-target organisms. Tsetse female survivors were found in large proportions of the dissected flies in the densely vegetated hotspot monitored during SAT. Given the observed proportion, it is not likely that these survivors were immigrant flies, all the more for the last 3 cycles, when supplementary spraying swaths would have necessitated a dispersal of more than 7.5 km within 2–3 days, which is highly unlikely given the dispersal rate of these species in this environment [29]. Actually, the probability to cross this distance would be almost null given the mean square displacements from 303 m/day to 780 m/day for *G. palpalis gambiensis*, and from 316 m/day to 775 m/day for *G. tachinoides*, as measured by mark-release-recapture experiments in similar landscapes in Burkina Faso and Mali [29]–[31]. Moreover, the tsetse observed densities along these tributaries were already low during the baseline survey [13]. Finally, target barriers deployed by the PATTEC Burkina Faso project along the tributaries coming from Burkina Faso into the target area (not shown in Figure 1) also reduced the probability that the observed non-teneral survivors were immigrant flies.

Trap efficiency is always very low and absence of capture does not mean absence of flies [32]. Considering a trap efficiency of 0.01 for biconical traps against these species, based on raw data collected in the area of Sideradougou, Burkina Faso [33], [34], and a mean width of the gallery forest of 100 m, the initial densities in the monitored section can be estimated at 2,783 flies/km^2^ of gallery forest [95%CI 1,733–4,467]. Given the simulation models [23], and for a 4-cycle treatment, the kill rate should be over 95% to warrant elimination, a suppression rate that was never attained here.

The spatial coverage of operations is another important factor to be considered when analysing the overall impact of interventions. Neither SAT nor the ancillary tools were applied across the whole project area. Operations, including aerial spraying, focused on the three main rivers, where suitable habitat for tsetse abounds. Whilst it is arguably a cost-effective approach to target interventions where they will have the largest impact on the tsetse populations, small pockets of flies located away from the main rivers may go untreated. The existence of these small pockets was not ruled out by the baseline data collection, which was limited to the main river systems [13]. The capture of both *G. tachinoides* and *G. palpalis gambiensis* in a right-hand tributary of the Kulpawn during the post-intervention survey supports the notion that small pockets of flies may be sustained through the dry season also in areas far from the main rivers. A similar situation was observed for *G. palpalis gambiensis* in dryer zones of its distribution area − for example in Senegal [35].

Although SAT failed to eliminate tsetse flies, the operations did reduce significantly tsetse abundance in a very short period of time. Considering the temperature in the study area (mean day temperature around 30°C), 4 cycles were required to cover the estimated PP. It was decided to limit SAT operations to a 4-cycle schedule because SAT-monitoring data (i.e. reduction rates and survival of mature females) showed that not even a fifth cycle would have achieved elimination, and it would have only increased the cost of suppression.

Importantly, suppression was achieved with few environmental side-effects. The impact of SAT operations on non-target aquatic, terrestrial and insectivorous fauna was studied and it will be presented in detail elsewhere. In essence, this environmental monitoring showed that the majority of non-target species were broadly unaffected [36].

If we consider the integrated control strategy as a whole, a number of factors must be called upon to explain the results of the post-intervention entomological survey. First, despite the deployment of barriers, possible re-invasion of flies from neighbouring areas may have occurred. In the absence of reinvasion barrier, *G. palpalis gambiensis* was estimated to reinvade at a speed of about 7.5 km/year along the Mouhoun river [30]. Moreover, both riverine tsetse species, and particularly *G. tachinoides*, are probably able to move between river basins [29], [37]. This is all the more of a preoccupation for *G. tachinoides*, which disperse ≈3 times faster than *G. palpalis gambiensis* in these fragmented landscapes. In the project area, not all barriers were in place before SAT operations started, and many were deployed during the spraying period. Models have shown that efficiency substantially increases if barriers are set up before the spraying cycles [23].

As far as ITC is concerned, the technique is efficient in reducing riverine tsetse populations [7], [38], [39] but arguably less so to achieve elimination, because a large proportion of riverine tsetse flies feed on alternative hosts [40], [41]. Theoretical models have shown that a daily mortality of 3% would eliminate a tsetse population [20], a results that would be obtained if only ≈10% of tsetse fed on ITC. However, Bauer et al. (1999) demonstrated in similar settings and using blood meal analyses, that small pockets of *G. tachinoides* were able to survive by feeding mainly on reptiles [38]. In fact, learning behaviour tends to protect flies that first fed on reptiles because they tend to feed repeatedly on the same host thereafter [40]. ITT, which can partially overcome this problem, could not be uniformly deployed within the project area, thus possibly contributing to the survival of pockets of tsetse. Moreover, these two techniques are very efficient at high tsetse densities, but less so when the abundance is low [8]. In the Loos Islands in Guinea, it was recently observed that, using the same techniques, decrease in tsetse density was sharp during the first months of the control campaign, but it required several years to obtain further sizable reductions in tsetse densities [9]; elimination has still not been achieved to date [42]. Looking at the overall integrated strategy implemented in Ghana by the “Multinational Project for the Creation of Sustainable Tsetse and Trypanosomosis Free Areas in East and West Africa”, we can conclude that it achieved a high level of suppression of tsetse populations using SAT as a key component. Baseline surveys had shown an average ADT of 6.51 (s.d. 26.59) and 0.14 (s.d. 0.69) for *G. tachinoides* and *G. p. gambiensis* respectively inside the spray blocks [13]. If we compare this with the data collected inside the spray blocks one year after SAT operations, we obtain relative reductions of ≈96% for both species. This result is interesting given the different ecologies of the flies in the target area, *G. tachinoides* favouring open riverine tickets whereas *G. p. gambiensis* preferring closed gallery forests, which are scarce in the study area [14]. Moreover, *G. tachinoides* feed more readily on cattle than *G. palpalis gambiensis* do [41], and might have been more impacted by ITC.

Further epidemiological and socio-economic studies are needed to assess the extent to which suppression of the tsetse populations resulted in a lower AAT risk and in a more conducive environment for livestock production.

Regarding the field costs for the operations, excluding salaries and depreciation of vehicles and equipment, they were $3,633,979, corresponding to $202/km^2^, including $3,000,000 for SAT operations (i.e. $445/km^2^ for suppressing tsetse in the 6,745 km^2^ sprayed), $200,000 for ground spraying, $133,890 for pour on treatments and $300,089 for impregnated targets (i.e. 35$/km^2^ for the integrated strategy used to maintain suppression during two years in the whole 18,000 km^2^ area). These costs are broadly in line with available estimates on the costs of tsetse and trypanosomosis control [43].

Tsetse suppression is being sustained in the Upper West Region, and schemes will be implemented to recover part of the cost from farmers who will be encouraged to use cost-effective techniques to protect their animals [44]. At the same time, suppression is planned to be extended to neighbouring Upper East and Northern regions. The regional strategy also foresees that on-going interventions in Western Burkina Faso will progressively expand their scope, targeting the areas at the border with Ghana, and thus reducing the cost of barriers.
